# Limited MOMP, ATM, and their roles in carcinogenesis and cancer treatment

**DOI:** 10.1186/s13578-020-00442-y

**Published:** 2020-06-18

**Authors:** Xuhui Bao, Xinjian Liu, Fang Li, Chuan-Yuan Li

**Affiliations:** 1 Department of Dermatology, Duke University Medical Center, Durham, NC USA; 2 School of Medicine, Sun Yat-Sen University, Guangzhou, China; 3 Department of Pharmacology and Cancer Biology, Duke University Medical Center, Durham, NC USA

## Abstract

Limited mitochondria outer membrane permeability (MOMP) is a novel biological process where mammalian cells initiate the intrinsic apoptosis pathway with increased mitochondrial permeability but survive. One of the major consequences of limited MOMP is apoptotic endonuclease-induced DNA double strand breaks. Recent studies indicate that these DNA double stand breaks and ensuing activation of DNA damage response factors such as ATM play important but previously underappreciated roles in carcinogenesis and tumor growth. Furthermore, novel non-canonical roles of DNA repair factors such as ATM in tumor growth and treatment are also emerging. In this review, we try to summarize recent findings on this newly revealed link between DNA double strand break repair and cell death pathways.

## The discovery of limited MOMP and its roles in cellular DNA double strand break induction and carcinogenesis

**Fig. 1:**
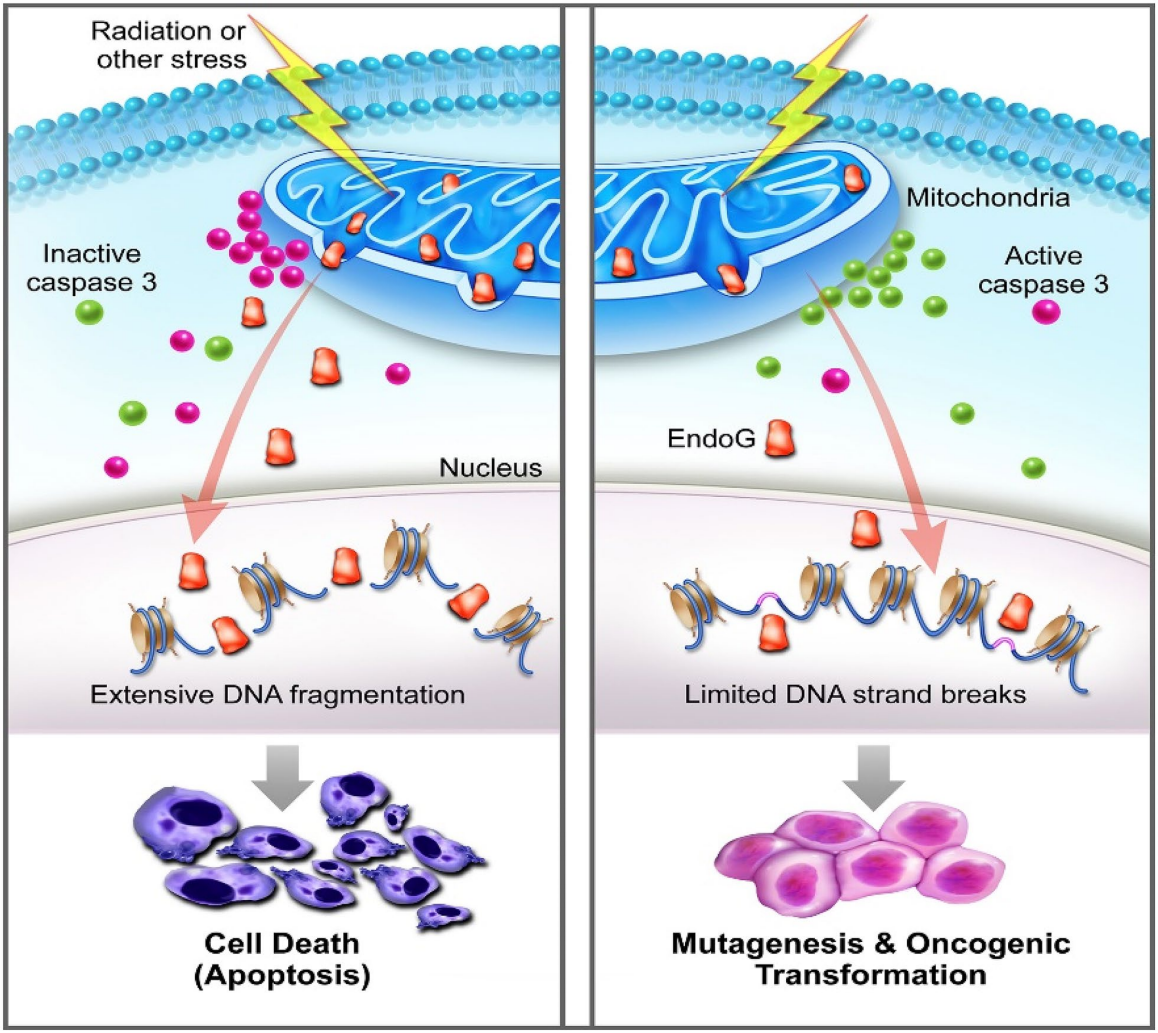
A schematic diagram illustrating how limited MOMP facilitates stress induced genetic instability and oncogenic transformation. Left panel shows the conventional scenario where mitochondrial permeability changes leads to activation of Casp3 and leakage of endonuclease G that kills the host cells. Right panel, on the other hand, shows partial leakage and survival of the cells with secondary genetic damage and oncogenic transformation (adapted from Liu et al. [19])

Mitochondria outer membrane permeability (MOMP) is a biological process initially described for cells undergoing programmed cell death or apoptosis. MOMP has been considered an essential process in the intrinsic pathway of apoptosis [1, 2]. In the classic paradigm, MOMP allows for cytochrome C leakage into the cytosol from the mitochondria, which stimulates APAF, the formation of the apoptosome [3], and subsequent activation of downstream apoptotic caspases such as Caspase 9, Caspase 3, and Caspase 7, which leads to destruction of critical cellular infrastructure and rapid cell death. However, in the past 10–15 years, it is becoming increasingly obvious MOMP and ensuing caspase activation does not always lead to apoptosis. In fact, our laboratory and others have shown that classical “apoptotic” caspases, including those involved in the execution of apoptosis such as Casp3 and Casp7, are involved in many non-cell death functions such as tissue regeneration [4] in Drosophila [5–8], hydra [9], and mouse [10]. They are also involved in embryonic stem cell differentiation [11, 12], and iPSC reprogramming [13]. Furthermore, they are involved in differentiation of somatic tissues such as those of T-cells [14, 15] and muscle cells [16, 17]. Not surprisingly, they are involved in cancer development [18]. More recently, our laboratory and others showed that sublethal caspase activation, caused by limited MOMP, occurred in murine and human cells exposed to ionizing radiation and DNA damaging chemicals [19, 20]. Moreover, in cells that experienced sublethal caspase activation, they experienced persistent DNA double strand breaks caused by apoptotic endonucleases such as CAD [21–23] (caspase-dependent DNase) and endoG [24, 25] (endonuclease G) and these double strand breaks played critical roles in malignant transformation both in vitro as well as in vivo [20] (Fig. 1).

In a separate study, we showed that myc-induced transformation of mammary epithelial cells depended on limited MOMP, i.e., sublethal Caspase 3 and endoG activation [26]. In fact, myc induced double strand break induction, which were shown to be involved in myc-induced genetic instability and transformation [27, 28], depended on Casp3 and endoG induction [26]. These findings therefore suggested that limited MOMP and sublethal activation of the apoptotic factors played critically important roles in radiation-, chemical-, and oncogene-induced carcinogenesis, roles that were previously unappreciated but may be key in understanding the carcinogenic process from a new perspective. Indeed, it also clarified some of the unanswered paradoxes in carcinogenesis. For example, myc is one of the few archetypical oncogenes identified. It is also one of the most powerful oncogenes. Very early on, it was discovered that myc was a potent inducer of apoptosis [29, 30]. On the other hand, apoptosis was known to be a process to eliminate damaged or unwanted cells, and a process to prevent carcinogenesis. In fact, p53, one of the most important tumor suppressor genes, is known to eliminate genetically unstable cells through apoptosis. How does one reconcile the powerful oncogenic properties of myc vs its potent apoptosis-inducing property? Our finding that myc’s ability to transform cells was mediated through limited MOMP, or sublethal activation of apoptotic caspases and endonuclease, provides a mechanistic explanation for this dilemma.

The realization that limited MOMP, through sublethal activation of apoptotic caspases and endonuclease, could generate DNA double strand breaks in the absence of any external insult not only deepened our understanding of how mammalian cells could generate DSBs indirectly, it also begs for additional questions on how the persistent DNA damage induced by limited MOMP affects the biology of tumor cells. This is because many tumor cells appear to possess persistent limited MOMP without any external insult, which leads to increased basal DNA DSB levels and activation of the DNA damage response (DDR) factors. Central among the DNA damage response factors is Ataxia Telangiectasia Mutated (*ATM*) [31], which plays critical roles in detecting and coordinating the repair of DNA double strand breaks, especially of those in the heterochromatin region [32].

## ATM as a central coordinator of DNA double strand break repair and its controversial roles in solid tumor development

The *ATM* gene was first cloned in 1995 [33]. It encodes a PI3K-related serine/threonine protein kinase (PIKK) to maintain genomic stability and integrity [33]. In the past few decades, ATM has been reported to play a central role in the repair of DNA double-strand breaks (DSB), from recognizing damaged DNA, to recruiting other repair proteins and regulating cell cycle arrests and facilitating apoptosis [31]. Upon sensing a DSB, ATM is activated and can phosphorylate a number of downstream effector proteins. The PIKK domain of ATM can recognize serine-glutamine and threonine-glutamine motifs of many other proteins, such as those of checkpoint kinase 1 (Chk1) and Chk2 to mediate cell-cycle checkpoint arrest; BRCA1 and RAD51 in DNA repair; p53 in apoptosis; protein kinase B (AKT) in cell survival; and KRAB-associated protein-1 (KAP1) in chromatin relaxation [31, 34–39]. Consequently, the network of ATM targets coordinates a number of signaling pathways in cellular response to DNA damage or genomic instability. Germ-line mutations in *ATM* can cause autosomal recessive inherited ataxia telangiectasia (A-T) syndrome, which exhibits a variety of manifestations including neurodegeneration, premature aging, extreme radio-sensitivity, metabolic disorders, and immune dysfunctions [31].

**Fig. 2:**
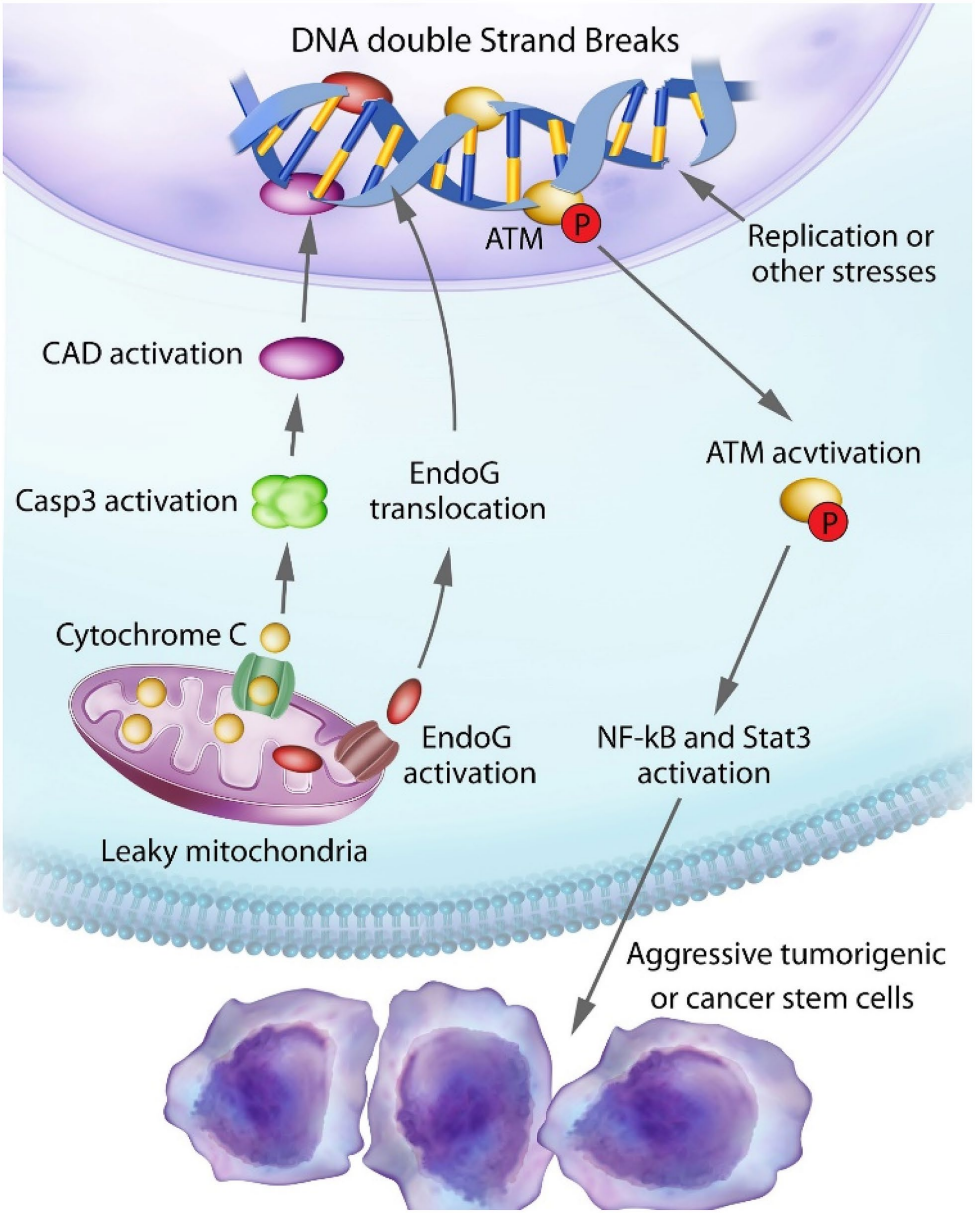
An illustration of our findings on spontaneous DNA double strand break induction and their roles in maintaining the stemness and tumorigenicity of cancer cells (adapted from Liu et al. [19])

Because of the role of ATM as a “guardian” of the genome, similar to that of the p53 protein, ATM has been traditionally designated as a tumor suppressor. Indeed, both murine and human carriers of ATM mutations are at a high risk for developing leukemia and lymphoma [40]. However, in terms of solid tumors, the evidence for a potential “driver” role for ATM is less obvious. There were some early evidence for heterozygous mutant ATM carriers possessing higher risk for breast cancer [41, 42]. However, other reports did not support such a role for ATM [43]. In fact studies conducted so far show that the only solid tissue malignancy that ATM heterozygote carriers have high risks for is breast cancer and the risk, if any, is moderate [44–46]. The fact that heterozygous ATM mutation carriers have only a slight increase in breast cancer risk and no increased risk for other solid tissue malignancies despite ATM’s profound influence on genomic instability is surprising. Such a discrepancy may indicate that ATM’s function in carcinogenesis is not straightforward as some of the other tumor suppressors such as p53 and Rb. Indeed, recent evidence suggest that ATM possesses non-canonical roles in promoting tumor growth and its activity is intimately associated with limited MOMP and caspase activation in cancer cells [47] (Fig. 2).

## ATM activation by limited MOMP and its role in promoting tumorigenicity of cancer cells

The existence of cancer stem cells (CSCs) is now well-recognized. CSCs are rare cells among cancer cells possessing enhanced ability to proliferate and form tumors [48–51]. In addition, similar to normal stem cells, they can also differentiation into lineage-specific subtype cells. CSCs have been identified in different malignancies by use of different cell surface makers such as CD133 for glioma [52] and CD24/CD44 for breast cancer [49]. In addition to their potent ability to form tumors, CSCs had increased resistance to cytotoxic chemotherapy and radiotherapy [53, 54], based on either their ability to pump out cytotoxic chemicals and/or increased capacity to repair DNA damage [53]. Despite numerous studies, there are some important questions in the field of CSC that remain unanswered: How are CSCs sustained? How do CSCs arise stochastically from non-stem cancer cells? Recent studies from our laboratory and others indicate that limited MOMP and activation of the DNA damage response plays a key role in maintaining the stem cell status of cancer cells [47]. In particular, we discovered many tumor cells have spontaneous, limited MOMP in the absence of external insults. Similar to cells exposed to stress, limited MOMP caused cytosolic leakage of cytochrome C and sublethal activation of apoptotic caspases (Casp3, 6, and 7) and endonucleases (CAD and endoG), which caused self-inflicted DSBs. The self-inflicted DSBs further caused activation of the DNA damage response (DDR), which included γH2AX foci formation and phosphorylation and activation of the ATM protein. Previously it was demonstrated that spontaneous occurring DSBs in cancer cells could occur because of replication stress generated by rapid proliferation and compromised cell cycle checkpoints in cancer cells [55, 56]. What are the relative contributions of replication stress vs limited MOMP and sublethal caspase activation? Our study showed, through CRISPR-mediated genetic knockout of apoptotic caspases, that limited MOMP was responsible for at least 50% DSBs, as determined by γH2AX foci staining [47].

What is the biological significance of self-inflicted DSBs by limited MOMP or replication stress? Our study showed that persistent activation of the DDR not only did not attenuate tumor cell growth, it enhanced tumor cells’ abilities to form colonies in 3D (i.e. growth in soft agar) [47]. Moreover, it boosted the tumor forming abilities of the cancer cells in vivo in mice. Genetic knockout of apoptotic caspases (Casp3/6/7) greatly attenuated the abilities of the host tumor cells to form tumors in vivo. Consistently, genetic knockout of apoptotic endonucleases, which are directly responsible for spontaneously occurring DSBs, also caused the slowdown in tumor growth. How do DSBs cause enhanced tumor growth? Our results showed that DDR activation, especially that of ATM activation, played a key role. In glioma cells, CD133+ glioma stem cells possessed higher levels of spontaneously induced DSBs and ATM phosphorylation. On the other hand, genetic knockout of ATM in human glioma cells showed significantly less expression of CD133, Oct4, Nestin, and activated STAT3, four important markers of glioma CSCs, suggesting that ATM activation were directly responsible for glioma CSC maintenance. Therefore, it appeared mechanistically ATM promotes stemness in glioma cells by phosphorylating and activating STAT3, which has been to be a crucial factor in glioma stem cells [57]. This was an important revelation, it explained not only on how CSCs are maintained but also provided a mechanism for CSCs to be generated de novo from non-CSC tumor cells through limited MOMP and activation of DDR, two important questions in cancer stem cell field.

Other studies have confirmed the importance of ATM in maintaining the stemness of breast cancer cells, albeit through different mechanisms. Valencia-González and colleagues also revealed that there was a significant expression of activated ATM only in the CSCs from Hela and human breast cancer MCF-7 cells, suggesting that phosphorylated ATM plays critical roles in breast cancer stem cells [58]. In another study, Antonelli et al. showed that ATM sustains breast cancer stem-like cells by promoting ATG4C expression and autophagy [59]. Taken together, these studies strongly suggest that ATM inhibition may be a promising strategy to suppress CSC properties and improve cancer treatment outcomes.

## Limited MOMP, caspase activation, and cellular innate immunity in cancer therapy

Very early on, inflammatory caspases were recognized as playing a central role in innate immunity. First identified among them was Casp1, which was not directly involved in apoptosis but rather controls the assembly of large multi-protein complexes called inflammasomes [60]. Various kinds inflammasomes, including NLRP3 inflammasome [61], the RIG-I inflammasome [62], and the AIM2 inflammasome [63, 64] are known to be engaged in antiviral innate immunity. Other caspases known to be involved in regulating inflammasomes include Caspases-4, -5, and -11, which can directly recognize lipopolysaccharides (LPS) [65–67]. More recently, the above caspases were also found to be involved in another form of programmed cell death called pyroptosis [68], which was first observed in *Shigella flexneri*-infected macrophages. Pyroptosis is now recognized as an important part of the organism’s innate immune system to get rid of pathogen-infected cells and to remove them by secondary phagocytes.

In recent years, it was reported classical apoptotic caspases, such as the intrinsic apoptotic caspases Casp-9,-3, and -7, were involved in negative regulation of cellular innate immunity and type I interferon induction in a manner that does not kill host cells. It was shown that these caspases played key roles in attenuating cytosolic mitochondrial DNA leakage or viral DNA or double stranded RNA-induced activation of cGAS/STING pathway [69, 70], often triggered during viral infections. Under these circumstances, caspases appear to dampen cGAS/STING of RIGI/MAVS pathway by inhibiting components of type I interferon signaling. Exact how apoptotic caspases carried out its suppression is not understood well. However, in some instances, apoptotic caspases appeared to directly cleave and inhibit cGAS, MAVS, or IRF3 to down-regulate Type I interferon production [71, 72].

Even though the regulation of cellular innate immunity by apoptotic caspases has been studied mostly from the perspective of controlling microbe infection, its importance in cancer treatment is almost self-evident given the importance of the immune system in surveillance and controlling tumor growth and in modulating the response of cancer treatments. In particular, the roles of cellular innate immunity, including these of the cGAS/STING pathway for cytosolic dsDNA detection and the RIG-I/MAVS pathway for cytosolic dsRNA detection are active areas of ongoing research in cancer immunotherapy, especially in the context of immune checkpoint blockade therapy (ICB) [73–77].

ICB therapy for cancer is rapidly gaining momentum as a major cancer treatment modality. This is because its use has profoundly improved the treatment outcome of some forms of cancer that previously had very poor prognosis [78]. These include lung [79–82], melanoma [83, 84], bladder cancers [82, 85, 86], and renal cell carcinoma [87]. Distinct from previously available forms of treatment such as classical cytotoxic treatment and targeted therapy, ICB therapy works in even patients with advanced diseases where other treatments have failed and can generate durable responses in a subset of patients. However, ICB treatment by itself only works for 10–30% of patients in those who receive it [88]. Therefore, there is much space for improvement for this revolutionary treatment.

Many research efforts have been devoted to understanding and improving ICB treatment. One of the key factors in determining whether a particular tumor responds to ICB treatment is the “hotness” of the tumor immune microenvironment (TIME) [89]. The “hotness” of TIME is mainly determined by the amount and quality of intratumoral lymphocyte infiltrate. In general, the more CD4+ or CD8+ T cells in the tumor mass, the better the tumor will respond to ICB treatment [90]. This is because ICB treatment mainly works by boosting the activities of anti-tumor T cells. It is now well-recognized there are two factors that can boost T cell numbers: (1) to increase the number of tumor-specific “neoantigens”, this is mainly determined by the number of tumor specific mutations; thus those tumors with high mutation rates, such as melanoma, lung cancer, bladder cancer, and a subset of colorectal cancer with microsatellite instability (MSI) respond well to ICB treatment [91]; (2) other tumor microenvironmental factors such as elevated cellular innate immunity that can boost intratumoral lymphocyte infiltration. While the number of neoantigens in a given tumor could not be easily manipulated, factors such cGAS/STING could be activated by external agents [74, 76, 77]. Indeed there are already clear preclinical data that suggest the essential role of cGAS/STING activation and downstream signaling with ICB therapy [73, 92, 93]. Furthermore, it was shown that the cGAS/STING pathway also played a key role in traditional therapies such as radiotherapy and cytotoxic chemotherapy.

Taken together, the fact that limited MOMP and caspases regulate cellular innate immunity and the latter is intimately involved in ICB therapy, cytotoxic chemotherapy, and radiotherapy suggest that there might exist opportunities to manipulate limited MOMP for therapeutic gain in cancer treatment. Examples of such manipulations include the inhibition of apoptotic caspases, which are the key mediators of MOMP. A recent report on the critical role of Casp9 in regulating murine tumor response to radiotherapy by regulating the cGAS/STING pathway in a cell autonomous manner provide strong evidence in this respect [94].

## ATM and its role in cellular innate immunity

What about ATM and the DNA damage response factors? Do they have any roles in regulating anti-tumor immunity? The evidence for ATM’s involvement in the immune system is actually quite complicated. Warren et al. showed that ATM is essential for the development of a protective immune memory against influenza A virus infection, suggesting that vaccination of A-T patients may not sufficiently protect them from the virus infection due to the malfunctioned immune system [95]. However, in cancer treatment, somatic ATM deficiency in cancer cells has a different manifestation. One of the most important ICB treatment target and predictive marker programmed death-ligand 1 (PD-L1) was found to be enriched in the gastric cancer with low expression of ATM in a clinical study [96]. Furthermore, Zhang et al. reported that silencing of ATM increased interferon signaling and boosted PD-L1 expression in vitro and in vivo in mouse pancreatic cancer models [97]. But other studies suggested that activation of ATM was correlated with PD-L1 upregulation in human bone osteosarcoma, lung cancer, and esophageal squamous cell carcinoma [98–100].

What do we know about ATM’s involvement in regulating innate immunity? It was previously reported that in *Drosophila*, ATM inhibition in glial cells activated the innate immune response to cause the death of glial cells and neurons [101]. Loss of ATM can also induce a proinflammatory innate immune response mediated by STING signaling in mouse microglial cells [102]. Elevated type I interferon (IFN) signaling was also detected in sera of A-T patients and *ATM*^−/−^ mice, resulting in enhanced innate immune response [103]. Indeed, it is possible that ATM deficiency, and in fact deficiencies in homologous recombination repair in general [104], may predict for better response to ICB treatment. Mechanistically this is very plausible because deficiencies in ATM or other homologous recombination factors such as BRCA1 or PARP1 may lead increased cytosolic dsDNA, which is more likely when cells are exposed to external insults such as radiotherapy, may activate the cGAS–STING pathway, which has clearly been demonstrated to play an active and essential role in the antitumor immunity, as shown in the previous section. These evidences thus suggest that targeting ATM may be a promising approach to enhance ICB treatment, especially in combination with radiotherapy and DNA damaging cytotoxic chemotherapy.

Because many of ATM’s biological roles depends on its kinase activities and ATM deficient cells are extremely sensitive to radiotherapy, there are many efforts to develop small molecule inhibitors to sensitize radiotherapy. One such inhibitor is AZD0156, which is an exceptionally potent and highly selective ATM inhibitor based on an imidazo[4,5-c]quinoline-2-one core. It showed excellent pharmacokinetics, a low predicted clinical dose, and a high maximum absorbable dose when orally administered at the preclinical level [105]. AZD0156 can inhibit ATM with the IC_50_ value of as low as 0.00004 µM and 0.00057 µM in enzyme and cell assays. It has been reported to significantly enhance the antitumor effect of irinotecan and olaparib in human colon and breast cancer xenograft models and well tolerated in animals [105]. AZD0156 is now being investigated as a monotherapy or in combination with either olaparib, cytotoxic chemotherapies, or novel anti-cancer agents to assess safety and tolerability in patients with advanced cancers (NCT02588105). AZD1390 is an enhanced version of AZD0156, specifically optimized to penetrate the blood–brain barrier (BBB), which has been confirmed in a preclinical monkey model [106]. It was also revealed that the free brain levels of AZD1390 peaked within 1 h after administration in a mouse orthotopic brain tumor model, and dissipated over a 24-h period, highly consistent with its activity of ATM inhibition [106]. When combined with radiotherapy, AZD1390 combination therapy significantly induced tumor regressions and prolonged animal survival compared to radiotherapy alone in syngeneic and xenograft glioma models as well as orthotopic lung-brain metastatic models [106]. A clinical trial to investigate AZD1390 is now recruiting patients to assess its safety and tolerability combined with radiotherapy in brain cancer patients (NCT03423628).

In summary, because of availability of clinical trial-ready small molecule compounds, it is very feasible to combine cytotoxic cancer therapy such as radiotherapy with ICB therapy and ATM inhibition. Such a “triple” threat approach may hold great promise in treating some forms of malignancies that are currently not responsive to ICB treatment alone.

## Conclusions

MOMP/apoptosis and DNA damage response are two fundamental biological processes previously thought to be well understood. However, it is evident from the literature that we covered in this review that each has numerous non-canonical and often times counter-intuitive functions. Recent studies on those non-canonical functions provided important new insights into the roles of those two processes in carcinogenesis and tumor response to treatment. Some of the insights may lead to the development of effective treatment/prevention strategies for cancer.

## Data Availability

Not applicable.
